# Crystal structure of *S*-hexyl (*E*)-3-(4-methoxy­benzyl­idene)di­thio­carbazate

**DOI:** 10.1107/S2056989015003199

**Published:** 2015-02-25

**Authors:** M. S. Begum, M. B. H. Howlader, R. Miyatake, E. Zangrando, M. C. Sheikh

**Affiliations:** a Department of Chemistry, Rajshahi University, Rajshahi-6205, Bangladesh; b Center for Environmental Conservation and Research Safety, University of Toyama, 3190 Gofuku, Toyama 930-8555, Japan; c Department of Chemical and Pharmaceutical Sciences, Via Giorgieri 1, 34127 Trieste, Italy; d Department of Applied Chemistry, Faculty of Engineering, University of Toyama, 3190 Gofuku, Toyama, 930-8555, Japan

**Keywords:** crystal structure, di­thio­carbazate, S-containing Schiff bases, hydrogen bonding

## Abstract

In the title compound, C_15_H_22_N_2_OS_2_, the di­thio­carbazate group adopts an *EE* conformation with respect to the C=N bond of the benzyl­idene moiety. The hexyl side chain adopts an extended conformation and the C—S—C—C torsion angle is −93.36 (13)°. In the crystal, inversion dimers linked by pairs of N—H⋯S hydrogen bonds generate *R*_2_^2^(8) loops.

## Related literature   

For a related structure and background references to Schiff bases, see: Howlader *et al.* (2015 ▸).
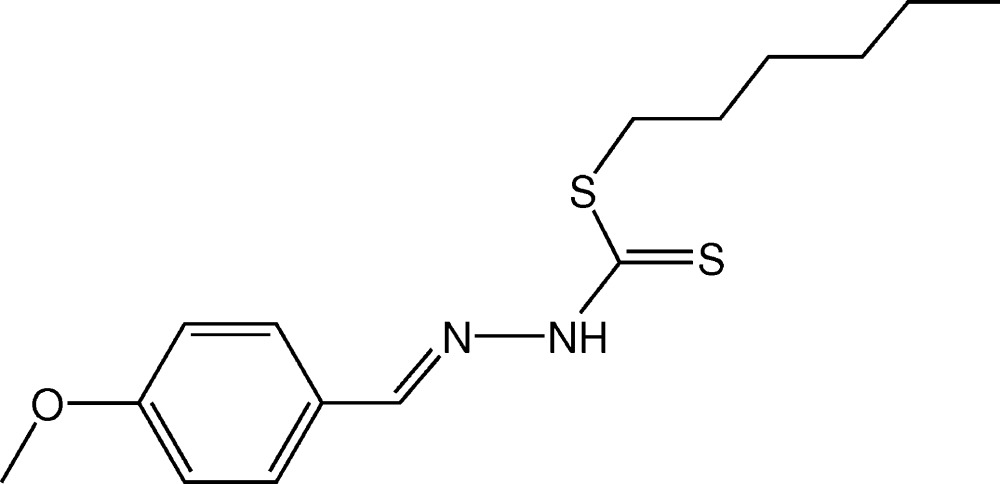


## Experimental   

### Crystal data  

C_15_H_22_N_2_OS_2_
*M* *_r_* = 310.47
Triclinic, 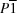

*a* = 4.55596 (8) Å
*b* = 12.4224 (3) Å
*c* = 14.9619 (3) Å
α = 75.7300 (9)°
β = 84.7599 (10)°
γ = 84.6141 (9)°
*V* = 814.99 (3) Å^3^
*Z* = 2
Cu *K*α radiation
μ = 2.93 mm^−1^
*T* = 173 K
0.29 × 0.26 × 0.17 mm

### Data collection  

Rigaku R-AXIS RAPID diffractometer
Absorption correction: multi-scan (*ABSCOR*; Rigaku, 1995 ▸) *T*_min_ = 0.350, *T*_max_ = 0.607
9377 measured reflections
2932 independent reflections
2385 reflections with *F* ^2^ > 2σ(*F* ^2^)
*R*_int_ = 0.082

### Refinement  

*R*[*F* ^2^ > 2σ(*F* ^2^)] = 0.051
*wR*(*F* ^2^) = 0.131
*S* = 1.08
2932 reflections
187 parameters
H atoms treated by a mixture of independent and constrained refinement
Δρ_max_ = 0.55 e Å^−3^
Δρ_min_ = −0.34 e Å^−3^

### 

Data collection: *RAPID-AUTO* (Rigaku, 2010 ▸); cell refinement: *RAPID-AUTO*; data reduction: *RAPID-AUTO*; program(s) used to solve structure: *SIR92* (Altomare *et al.*, 1994 ▸); program(s) used to refine structure: *SHELXL97* (Sheldrick, 2008 ▸); molecular graphics: *CrystalStructure* (Rigaku, 2010 ▸); software used to prepare material for publication: *CrystalStructure*.

## Supplementary Material

Crystal structure: contains datablock(s) General, I. DOI: 10.1107/S2056989015003199/hb7372sup1.cif

Structure factors: contains datablock(s) I. DOI: 10.1107/S2056989015003199/hb7372Isup2.hkl

Supporting information file. DOI: 10.1107/S2056989015003199/hb7372Isup3.cml

**Fig. 1.**
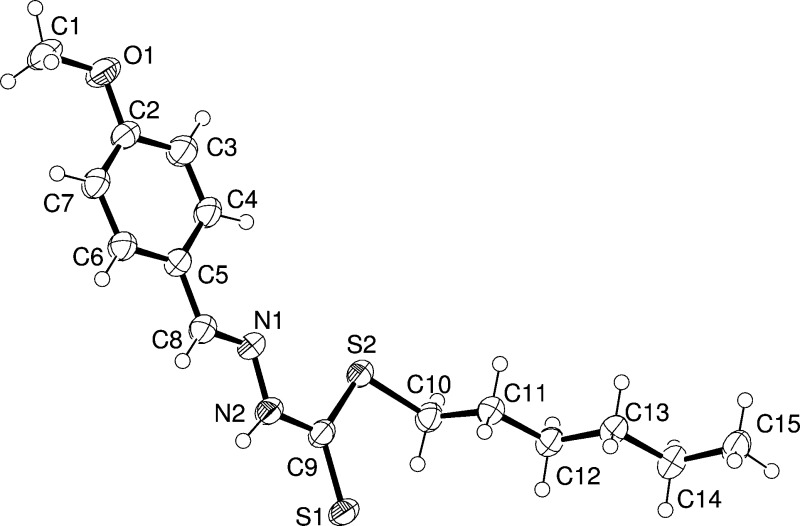
ORTEP drawing (ellipsoid probability at 50%) of molecule (1).

ORTEP . DOI: 10.1107/S2056989015003199/hb7372fig1.tif
*ORTEP* drawing (ellipsoid probability at 50%) of mol­ecule (1).

**Fig. 2.**
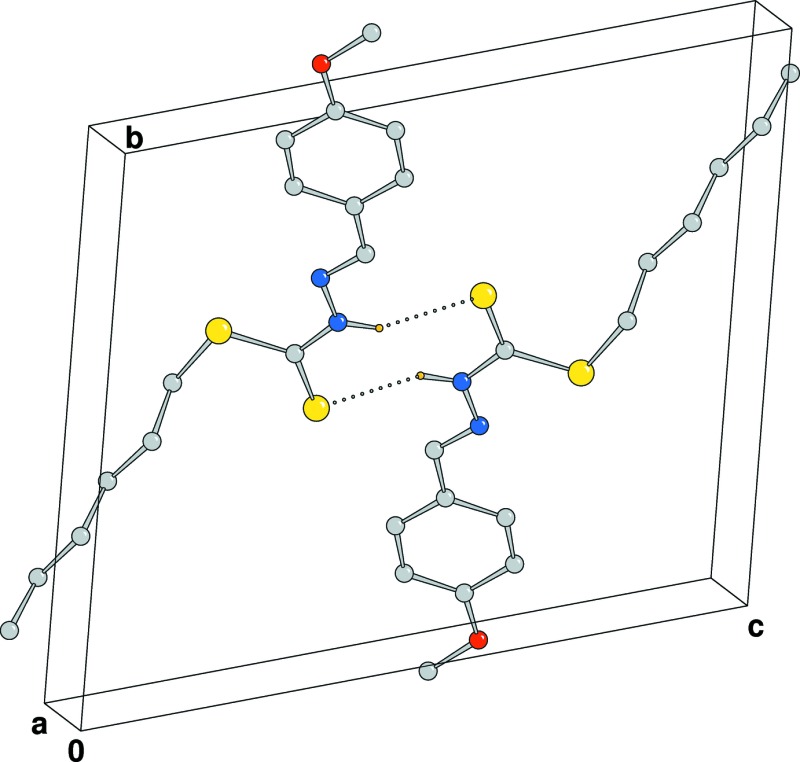
Crystal packing of (1) showing pair of molecules connected by N—H..·S interactions (dashed lines). H atoms not involved in hydrogen bonding have been omitted.

. DOI: 10.1107/S2056989015003199/hb7372fig2.tif
Crystal packing of (1) showing pair of mol­ecules connected by N—H..·S inter­actions (dashed lines). H atoms not involved in hydrogen bonding have been omitted.

CCDC reference: 1044475

Additional supporting information:  crystallographic information; 3D view; checkCIF report

## Figures and Tables

**Table 1:** Hydrogen-bond geometry (, )

*D*H*A*	*D*H	H*A*	*D* *A*	*D*H*A*
N2H9S1^i^	0.92(3)	2.51(3)	3.3614(18)	154(2)

Symmetry code: (i) 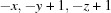.

## supplementary crystallographic information

### S1. Chemical context

As part of our ongoing structural studies of S-containing Schiff bases (Howlader *et al.*, 2015), we now describe the structure of the title compound.

### S2. Structural commentary

The molecule of the title compound is shown in Fig. 1. The Schiff base exists in thione tautomeric form with the di­thio­carbazate fragment adopting an EE configuration with respect to the C=N bond of the benzyl­idene moiety. The *β*-nitro­gen and the thio­keto sulphur are *trans* located with respect to the C(9)—N(2) bond. With the exception of the S-hexyl chain the molecule shows co-planar atoms indicating an electron delocalization within it. The bond lengths and angles are close comparable to those detected in the S-hexyl (*E*)-3-(4-methyl­benzyl­idene)di­thio­carbazate (Howlader *et al.*, 2015) which differs for a methyl replacing the meth­oxy group -O(1)—CH_3_. However a different conformation is exhibited by the hexyl chain in the two molecules likely induced by packing requirements. In fact the torsion angle S(2)—C(10)—C(11)—C(12) is of 173.99 (13) and 66.61 (1)° in the present complex and in the methyl derivative, respectively.

### S3. Supra­molecular features

The crystal packing evidences the ligand molecules piled along axis *a* and connected in pair by N2—H···S1 hydrogen bonds (H···S = 2.51 (3) Å, N···S = 3.361 (2) Å, O–H···S angle = 154 (2)°) as shown in Fig. 2. On the other hand no appreciable π–π inter­action among aromatic rings is present in the crystal packing.

### S4. Synthesis and crystallization

To an ethano­lic solution of KOH (2.81 g, 0.05 mol) hydrazine hydrate (2.50 g, 0.05 mol, 99%) was added and the mixture was stirred at 273 K. To this solution carbon di­sulfide (3.81 g, 0.05 mol) was added dropwise with constant stirring for one hour. Then *n*-bromo­hexane (8.25 g, 0.05 mol) was added dropwise with vigorous stirring at 273 K for an additional hour. Finally, 4-meth­oxy­benzaldehyde (6.81 g, 0.05 mol) in ethanol was added and the mixture refluxed for 30 min. The mixture was filtered while hot and then the filtrate was cooled to 273 K giving a precipitate of the Schiff base product. It was recrystallized from ethanol at room temperature and dried in a vacuum desiccator over anhydrous CaCl_2_. Colourless blocks of the title compound were obtained by slow evaporation of an ethanol/chloro­form (2:1) solution after 29 days (m.p. 369 K).

### S5. Refinement

Crystal data, data collection and structure refinement details are summarized in Table 1. Hydrogen atoms were located geometrically and treated as riding atoms with C—H = 0.95–0.99 Å and *U*_ĩso_(H) = 1.2*U*_eq_(C). The hydrogen atom at N2 was detected on the difference Fourier map and freely refined.

### Crystal data

**Table:**

C_15_H_22_N_2_OS_2_	*Z* = 2
*M**_r_* = 310.47	*F*(000) = 332.00
Triclinic, *P*1	*D*_x_ = 1.265 Mg m^−^^3^
Hall symbol: -P 1	Cu *K*α radiation, λ = 1.54187 Å
*a* = 4.55596 (8) Å	Cell parameters from 8917 reflections
*b* = 12.4224 (3) Å	θ = 3.1–68.2°
*c* = 14.9619 (3) Å	µ = 2.93 mm^−^^1^
α = 75.7300 (9)°	*T* = 173 K
β = 84.7599 (10)°	Prism, colorless
γ = 84.6141 (9)°	0.29 × 0.26 × 0.17 mm
*V* = 814.99 (3) Å^3^	

### Data collection

**Table:**

Rigaku R-AXIS RAPID diffractometer	2385 reflections with *F*^2^ > 2σ(*F*^2^)
Detector resolution: 10.000 pixels mm^-1^	*R*_int_ = 0.082
ω scans	θ_max_ = 68.2°
Absorption correction: multi-scan (*ABSCOR*; Rigaku, 1995)	*h* = −5→5
*T*_min_ = 0.350, *T*_max_ = 0.607	*k* = −14→14
9377 measured reflections	*l* = −17→17
2932 independent reflections	

### Refinement

**Table:**

Refinement on *F*^2^	Secondary atom site location: difference Fourier map
*R*[*F*^2^ > 2σ(*F*^2^)] = 0.051	Hydrogen site location: inferred from neighbouring sites
*wR*(*F*^2^) = 0.131	H atoms treated by a mixture of independent and constrained refinement
*S* = 1.08	*w* = 1/[σ^2^(*F*_o_^2^) + (0.070*P*)^2^] where *P* = (*F*_o_^2^ + 2*F*_c_^2^)/3
2932 reflections	(Δ/σ)_max_ = 0.001
187 parameters	Δρ_max_ = 0.55 e Å^−^^3^
0 restraints	Δρ_min_ = −0.34 e Å^−^^3^
Primary atom site location: structure-invariant direct methods	

### Special details

**Table:**

Geometry. ENTER SPECIAL DETAILS OF THE MOLECULAR GEOMETRY
Refinement. Refinement was performed using all reflections. The weighted *R*-factor (*wR*) and goodness of fit (*S*) are based on *F*^2^. *R*-factor (gt) are based on *F*. The threshold expression of *F*^2^ > 2.0 σ(*F*^2^) is used only for calculating *R*-factor (gt).

### Fractional atomic coordinates and isotropic or equivalent isotropic displacement parameters (Å^2^)

**Table:**

	*x*	*y*	*z*	*U*_iso_*/*U*_eq_	
S1	−0.15957 (13)	0.58111 (4)	0.61120 (3)	0.0472 (3)	
S2	0.02305 (12)	0.40174 (4)	0.77974 (3)	0.0394 (2)	
O1	1.3023 (4)	−0.11118 (12)	0.72987 (10)	0.0557 (5)	
N1	0.4025 (4)	0.31956 (12)	0.65318 (11)	0.0372 (5)	
N2	0.2334 (4)	0.41308 (13)	0.61147 (12)	0.0397 (5)	
C1	1.4937 (6)	−0.16105 (19)	0.66823 (17)	0.0612 (7)	
C2	1.1429 (5)	−0.01440 (15)	0.69352 (14)	0.0411 (6)	
C3	0.9556 (6)	0.03120 (17)	0.75560 (14)	0.0512 (7)	
C4	0.7806 (5)	0.12633 (16)	0.72645 (14)	0.0437 (6)	
C5	0.7833 (5)	0.18084 (15)	0.63242 (13)	0.0360 (5)	
C6	0.9755 (5)	0.13648 (16)	0.57096 (13)	0.0410 (5)	
C7	1.1522 (5)	0.03933 (17)	0.60045 (14)	0.0416 (6)	
C8	0.5930 (5)	0.28016 (16)	0.59886 (14)	0.0386 (5)	
C9	0.0392 (5)	0.46598 (15)	0.66220 (13)	0.0361 (5)	
C10	−0.2390 (5)	0.49416 (16)	0.82844 (13)	0.0383 (5)	
C11	−0.0961 (5)	0.58123 (16)	0.86125 (13)	0.0380 (5)	
C12	−0.3195 (5)	0.65013 (15)	0.91114 (13)	0.0371 (5)	
C13	−0.1786 (5)	0.72953 (16)	0.95358 (14)	0.0389 (5)	
C14	−0.3987 (5)	0.80105 (16)	1.00112 (15)	0.0443 (6)	
C15	−0.2532 (5)	0.87615 (16)	1.04683 (15)	0.0478 (6)	
H1	1.6361	−0.1081	0.6350	0.0734*	
H2	1.3769	−0.1813	0.6238	0.0734*	
H3	1.6000	−0.2281	0.7035	0.0734*	
H4	0.9496	−0.0047	0.8196	0.0615*	
H5	0.6555	0.1561	0.7702	0.0525*	
H6	0.9861	0.1736	0.5073	0.0492*	
H7	1.2792	0.0096	0.5571	0.0499*	
H8	0.6098	0.3169	0.5351	0.0464*	
H9	0.238 (6)	0.437 (2)	0.5482 (18)	0.066 (8)*	
H10	−0.3774	0.5321	0.7812	0.0460*	
H11	−0.3559	0.4493	0.8812	0.0460*	
H12	0.0025	0.6316	0.8074	0.0455*	
H13	0.0573	0.5440	0.9036	0.0455*	
H14	−0.4602	0.6934	0.8667	0.0445*	
H15	−0.4335	0.5989	0.9605	0.0445*	
H16	−0.0596	0.7791	0.9044	0.0467*	
H17	−0.0421	0.6859	0.9993	0.0467*	
H18	−0.5293	0.8476	0.9549	0.0532*	
H19	−0.5239	0.7518	1.0485	0.0532*	
H20	−0.1348	0.8306	1.0959	0.0574*	
H21	−0.4057	0.9225	1.0735	0.0574*	
H22	−0.1251	0.9241	1.0007	0.0574*	

### Atomic displacement parameters (Å^2^)

**Table:**

	*U*^11^	*U*^22^	*U*^33^	*U*^12^	*U*^13^	*U*^23^
S1	0.0602 (5)	0.0390 (4)	0.0377 (4)	0.0190 (3)	−0.0040 (3)	−0.0083 (3)
S2	0.0510 (4)	0.0320 (3)	0.0342 (3)	0.0070 (3)	−0.0034 (3)	−0.0094 (2)
O1	0.0693 (12)	0.0408 (9)	0.0489 (9)	0.0207 (8)	−0.0009 (8)	−0.0058 (7)
N1	0.0397 (11)	0.0302 (9)	0.0415 (10)	0.0059 (8)	−0.0050 (8)	−0.0104 (7)
N2	0.0460 (12)	0.0356 (9)	0.0352 (10)	0.0096 (8)	−0.0016 (8)	−0.0092 (8)
C1	0.0696 (18)	0.0483 (13)	0.0608 (15)	0.0272 (13)	−0.0060 (13)	−0.0151 (11)
C2	0.0467 (14)	0.0316 (11)	0.0433 (12)	0.0069 (10)	−0.0043 (10)	−0.0091 (9)
C3	0.0699 (18)	0.0436 (12)	0.0353 (11)	0.0114 (12)	−0.0014 (11)	−0.0068 (9)
C4	0.0531 (15)	0.0405 (12)	0.0363 (11)	0.0092 (11)	0.0019 (10)	−0.0132 (9)
C5	0.0366 (13)	0.0328 (10)	0.0393 (11)	0.0018 (9)	−0.0032 (9)	−0.0115 (9)
C6	0.0461 (14)	0.0402 (11)	0.0345 (11)	0.0060 (10)	0.0003 (9)	−0.0096 (9)
C7	0.0436 (14)	0.0405 (11)	0.0399 (11)	0.0073 (10)	0.0018 (9)	−0.0140 (9)
C8	0.0422 (13)	0.0366 (11)	0.0368 (11)	0.0034 (10)	−0.0015 (9)	−0.0110 (9)
C9	0.0384 (12)	0.0292 (10)	0.0412 (11)	0.0023 (9)	−0.0026 (9)	−0.0110 (8)
C10	0.0418 (13)	0.0363 (11)	0.0363 (10)	0.0015 (10)	0.0017 (9)	−0.0112 (9)
C11	0.0404 (13)	0.0358 (10)	0.0380 (11)	0.0028 (10)	−0.0024 (9)	−0.0117 (9)
C12	0.0390 (13)	0.0329 (10)	0.0386 (11)	0.0003 (10)	0.0017 (9)	−0.0097 (9)
C13	0.0404 (13)	0.0331 (10)	0.0432 (11)	0.0019 (10)	−0.0015 (9)	−0.0115 (9)
C14	0.0461 (14)	0.0356 (11)	0.0521 (13)	−0.0006 (10)	0.0029 (11)	−0.0155 (10)
C15	0.0592 (16)	0.0336 (11)	0.0517 (13)	−0.0005 (11)	−0.0003 (11)	−0.0148 (10)

### Geometric parameters (Å, º)

**Table:**

S1—C9	1.6713 (19)	C1—H1	0.980
S2—C9	1.7408 (19)	C1—H2	0.980
S2—C10	1.809 (2)	C1—H3	0.980
O1—C1	1.424 (3)	C3—H4	0.950
O1—C2	1.362 (3)	C4—H5	0.950
N1—N2	1.377 (2)	C6—H6	0.950
N1—C8	1.281 (3)	C7—H7	0.950
N2—C9	1.343 (3)	C8—H8	0.950
C2—C3	1.391 (3)	C10—H10	0.990
C2—C7	1.387 (3)	C10—H11	0.990
C3—C4	1.362 (3)	C11—H12	0.990
C4—C5	1.402 (3)	C11—H13	0.990
C5—C6	1.389 (3)	C12—H14	0.990
C5—C8	1.449 (3)	C12—H15	0.990
C6—C7	1.385 (3)	C13—H16	0.990
C10—C11	1.512 (4)	C13—H17	0.990
C11—C12	1.529 (3)	C14—H18	0.990
C12—C13	1.513 (4)	C14—H19	0.990
C13—C14	1.522 (3)	C15—H20	0.980
C14—C15	1.514 (4)	C15—H21	0.980
N2—H9	0.92 (3)	C15—H22	0.980
			
C9—S2—C10	102.71 (9)	C7—C6—H6	119.260
C1—O1—C2	117.93 (16)	C2—C7—H7	120.107
N2—N1—C8	115.16 (16)	C6—C7—H7	120.106
N1—N2—C9	120.57 (16)	N1—C8—H8	119.244
O1—C2—C3	116.35 (17)	C5—C8—H8	119.248
O1—C2—C7	124.76 (19)	S2—C10—H10	108.855
C3—C2—C7	118.89 (18)	S2—C10—H11	108.851
C2—C3—C4	121.27 (18)	C11—C10—H10	108.851
C3—C4—C5	120.7 (2)	C11—C10—H11	108.850
C4—C5—C6	117.88 (17)	H10—C10—H11	107.702
C4—C5—C8	121.99 (19)	C10—C11—H12	109.133
C6—C5—C8	120.13 (17)	C10—C11—H13	109.138
C5—C6—C7	121.48 (17)	C12—C11—H12	109.128
C2—C7—C6	119.8 (2)	C12—C11—H13	109.124
N1—C8—C5	121.51 (17)	H12—C11—H13	107.854
S2—C9—S1	126.46 (13)	C11—C12—H14	108.871
S2—C9—N2	113.32 (13)	C11—C12—H15	108.874
S1—C9—N2	120.22 (14)	C13—C12—H14	108.869
S2—C10—C11	113.57 (15)	C13—C12—H15	108.863
C10—C11—C12	112.36 (17)	H14—C12—H15	107.709
C11—C12—C13	113.50 (17)	C12—C13—H16	108.730
C12—C13—C14	114.11 (18)	C12—C13—H17	108.731
C13—C14—C15	113.33 (19)	C14—C13—H16	108.724
N1—N2—H9	120.7 (15)	C14—C13—H17	108.719
C9—N2—H9	118.5 (15)	H16—C13—H17	107.637
O1—C1—H1	109.480	C13—C14—H18	108.903
O1—C1—H2	109.467	C13—C14—H19	108.907
O1—C1—H3	109.466	C15—C14—H18	108.906
H1—C1—H2	109.470	C15—C14—H19	108.918
H1—C1—H3	109.471	H18—C14—H19	107.733
H2—C1—H3	109.473	C14—C15—H20	109.466
C2—C3—H4	119.366	C14—C15—H21	109.469
C4—C3—H4	119.363	C14—C15—H22	109.479
C3—C4—H5	119.668	H20—C15—H21	109.471
C5—C4—H5	119.669	H20—C15—H22	109.465
C5—C6—H6	119.260	H21—C15—H22	109.476
			
C9—S2—C10—C11	−93.36 (13)	C2—C3—C4—C5	0.4 (4)
C10—S2—C9—S1	−2.95 (18)	C3—C4—C5—C6	−1.8 (4)
C10—S2—C9—N2	177.54 (14)	C3—C4—C5—C8	178.0 (2)
C1—O1—C2—C3	179.23 (18)	C4—C5—C6—C7	2.3 (3)
C1—O1—C2—C7	0.2 (3)	C4—C5—C8—N1	−3.6 (4)
N2—N1—C8—C5	−178.88 (17)	C6—C5—C8—N1	176.24 (19)
C8—N1—N2—C9	−175.79 (17)	C8—C5—C6—C7	−177.54 (18)
N1—N2—C9—S2	−1.8 (3)	C5—C6—C7—C2	−1.3 (4)
N1—N2—C9—S1	178.61 (15)	S2—C10—C11—C12	−173.99 (10)
O1—C2—C3—C4	−178.38 (19)	C10—C11—C12—C13	173.87 (13)
O1—C2—C7—C6	178.73 (19)	C11—C12—C13—C14	178.35 (13)
C3—C2—C7—C6	−0.2 (4)	C12—C13—C14—C15	177.40 (13)
C7—C2—C3—C4	0.7 (4)		

### Hydrogen-bond geometry (Å, º)

**Table:**

*D*—H···*A*	*D*—H	H···*A*	*D*···*A*	*D*—H···*A*
N2—H9···S1^i^	0.92 (3)	2.51 (3)	3.3614 (18)	154 (2)

Symmetry code: (i) −*x*, −*y*+1, −*z*+1.
