# Supplementary figures and images for: Crystal structure of S-hexyl (E)-3-(4-methoxy­benzyl­idene)di­thio­carbazate
Source: Acta Crystallogr E Crystallogr Commun. 2015 Feb 25;71(Pt 3):o199. doi: 10.1107/S2056989015003199 (PMC4350723; doi:10.1107/S2056989015003199)

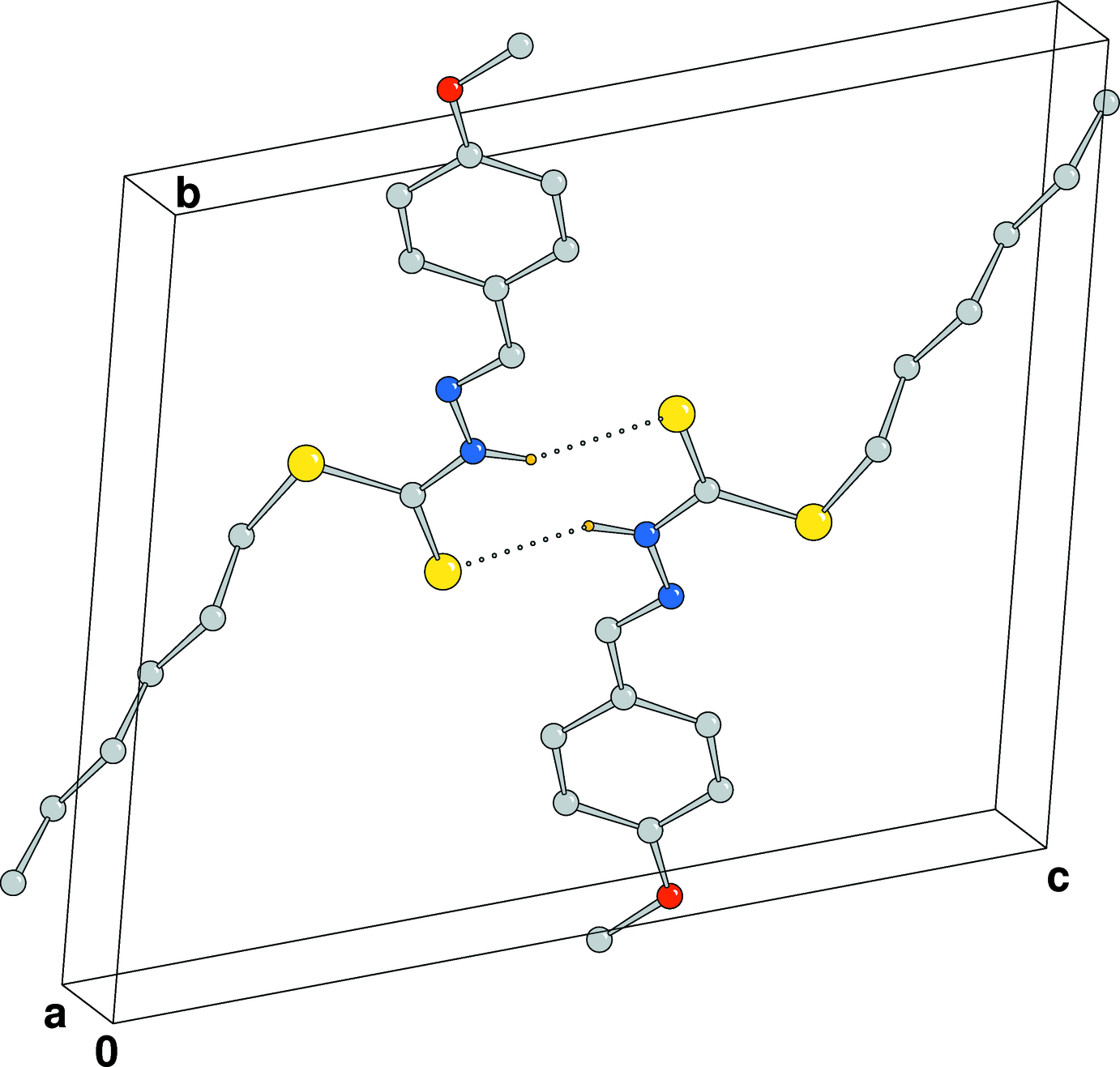

Supplement: Supplementary file 5 [file e-71-0o199-fig2.tif]
